# A case of AML with complex karyotype and chromosomal rearrangement of KMT2A

**DOI:** 10.1002/ccr3.9139

**Published:** 2024-07-22

**Authors:** Habib Moshref Razavi, Agata Minor

**Affiliations:** ^1^ Division of Hematopathology Royal Columbian Hospital New Westminster British Columbia Canada; ^2^ Department of Pathology and Laboratory Medicine University of British Columbia Vancouver British Columbia Canada; ^3^ Department of Cytogenetics the Royal Columbian hospital, Fraser Health Authority New Westminster British Columbia Canada

**Keywords:** acute myeloid leukemia, Auer rods, complex karyotype, KMT2A rearrangement

## Abstract

This is a case of an elderly patient diagnosed with acute myeloid leukemia (AML). While morphological findings, including numerous long, slender, cigar‐shaped Auer rods, suggested AML with t(8;21), cytogenetic and FISH analysis revealed abnormalities in chromosome 11 and the KMT2A (MLL) gene. The patient also exhibited double minutes, typically seen in AML and linked to a complex karyotype and poor prognosis. Correlating morphological findings with molecular genetics and cytogenetics is crucial for accurate diagnosis and treatment.

## 1 CASE DESCRIPTION

A 78‐year‐old female with dementia, type 2 diabetes mellitus, and a remote history of ovarian cancer treated with oophorectomy followed by chemotherapy, as well as stage IV lymphoma (2002), previously treated with partial gastrectomy and chemotherapy, was referred for pancytopenia (hemoglobin at 97 g/L, platelets at 13 × 10^9^/L, and neutrophils 0.2 × 10^9^/L). She experienced a rapid deterioration of her counts since March 2023, a time when they were previously normal. At the time of admission, her vitamin B12 levels were nonexistent, and she was supplemented to normal levels without affecting the correction of cytopenias.

**FIGURE 1:**
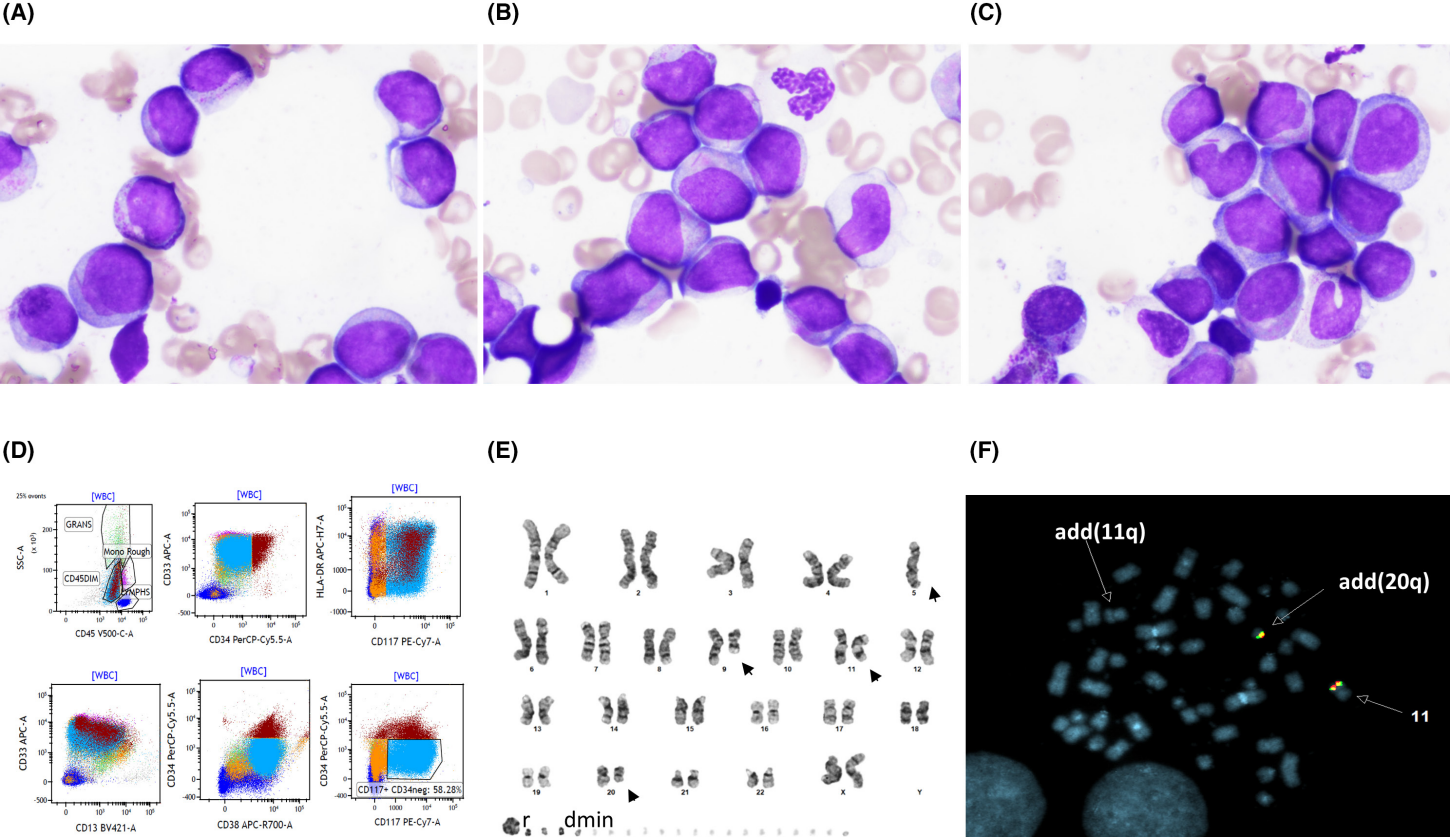
A case of acute myeloid leukemia featuring long thin Auer rods and occasional Chidiack Highashi type granules is noted (Panels A–C). Flow cytometry showed blasts with low to intermediate side scatter, expressing dim CD45, partial CD34, CD117, HLA‐DR, CD13, CD33, CD38, CD7, CD15, dim CD4, CD64, partial CD11b, and cMPO (Panel D). Karyogram shows a complex karyotype, a ring chromosome and between 7 and 58 double minute (panel E). Analysis of 10 abnormal metaphase cells showed one KMT2A fusion signal on the normal chromosome 11 and one KMT2A fusion signal on the add(20q). There was no KMT2A hybridization signal on the add(11q). These results show that there is no deletion or rearrangement of KMT2A; however, KMT2A has been moved to the add(20) (FISH results, Panel F).

As part of her workup, a bone marrow aspirate and biopsy highlighted a large blast infiltrate (63.4%), with blast cells occasionally presenting long, thin/slender, sometimes cigar‐shaped Auer rods (Figure 1. Panels A–C, May Grunwald‐Giemsa ×50 magnification). Flow cytometry showed blasts with low to intermediate side scatter, expressing dim CD45, partial CD34, CD117, HLA‐DR, CD13, CD33, CD38, CD7, CD15, dim CD4, CD64, partial CD11b, and cMPO (Figure 1. selected plots, panel D). Karyogram analysis of 15 metaphase cells uncovered a complex clone with chromosomal abnormalities, such as the loss of one copy of chromosome 5, a deletion on chromosome 9 (q12 to q31‐33), and additional material attached to chromosomes 11 and 20 (q23 and q11.2, respectively). A ring chromosome and between 7 and 58 double minutes were observed in each metaphase cell (Figure 1. Panel E).

FISH analysis was performed with the KMT2A dual‐color, break‐apart probe (purchased from Metasystems) to determine if the KMT2A gene located on 11q23 was either deleted or rearranged. Analysis of 10 abnormal metaphase cells showed one KMT2A fusion signal on the normal chromosome 11 and one KMT2A fusion signal on the add(20q). There was no KMT2A hybridization signal on the add(11q). These results show that there is no deletion or rearrangement of KMT2A; however, KMT2A has been moved to the add(20) (Figure 1. Panel F).

As there was suspicion of a t(8;21) involving RUNX1T1/RUNX1, FISH analysis was performed with the RUNX1T1/RUNX1 dual‐color, dual‐fusion probe set (purchased from Vysis) that binds to RUNX1T1 on 8q21.3 and to RUNX1 on 21q22. This study shows no evidence of the t(8;21) fusion on analysis of 10 abnormal metaphase cells (not shown).

In summary, in this patient, a complex abnormal clone associated with an adverse prognosis in AML is seen.^1^ FISH analysis did not show a rearrangement of KMT2A; however, KMT2A was moved to the long arm of the add(20). Therefore, the add(20) contains chromosome 11 material that includes at least KMT2A. Double minutes represent extrachromosomal gene amplification, most commonly of MYC and less frequently of KMT2A (previously called MLL).^2^ The double minutes observed in this patient did not show hybridization with the KMT2A probe; therefore, their origin remains unknown. FISH with the MYC probe was not performed. Double minutes have been primarily observed in AML and have been associated with a complex karyotype, as observed in this patient, and a poor patient outcome.^2^ The patient is transfusion‐dependent but is overall faring well post her fourth round of azacitidine therapy.

## AUTHOR CONTRIBUTIONS

**Habib Moshref Razavi:** Conceptualization; formal analysis; project administration; supervision; writing – original draft; writing – review and editing. **Agata Minor:** Conceptualization; data curation; formal analysis; writing – original draft; writing – review and editing.

## 2 FUNDING INFORMATION

There are no fundings associated wtih this article.

## CONSENT

Written informed consent was obtained from the patient to publish this report in accordance with the journal's patient consent policy.

## Data Availability

All data are available for review.
